# Acoustic emissions and kinematic instability of the osteoarthritic knee joint: comparison with radiographic findings

**DOI:** 10.1038/s41598-021-98945-2

**Published:** 2021-10-01

**Authors:** Mika T. Nevalainen, Olli Veikkola, Jerome Thevenot, Aleksei Tiulpin, Jukka Hirvasniemi, Jaakko Niinimäki, Simo S. Saarakkala

**Affiliations:** 1 Department of Diagnostic Radiology, Oulu University Hospital, P.O. Box 50, 90029 Oulu, Finland; 2 Medical Research Center Oulu, University of Oulu, P.O. Box 8000, Oulu, Finland; 3 Research Unit of Medical Imaging, Physics and Technology, Faculty of Medicine, University of Oulu, POB 5000, 90014 Oulu, Finland; 4 Department of Radiology and Nuclear Medicine, Erasmus MC, University Medical Center, P.O. Box 2040, 3000 CA Rotterdam, The Netherlands

**Keywords:** Diagnostic markers, Predictive markers, Osteoarthritis, Musculoskeletal system, Biomedical engineering, Imaging techniques

## Abstract

To evaluate the acoustic emissions (AE) and kinematic instability (KI) of the osteoarthritic (OA) knee joints, and to compare these signals to radiographic findings. Sixty-six female and 43 male participants aged 44–67 were recruited. On radiography, joint-space narrowing, osteophytes and Kellgren–Lawrence (KL) grade were evaluated. Based on radiography, 54 subjects (the study group) were diagnosed with radiographic OA (KL-grade ≥ 2) while the remaining 55 subjects (KL-grade < 2) formed the control group. AE and KI were recorded with a custom-made prototype and compared with radiographic findings using area-under-curve (AUC) and independent T-test. Predictive logistic regression models were constructed using leave-one-out cross validation. In females, the parameters reflecting consistency of the AE patterns during specific tasks, KI, BMI and age had a significant statistical difference between the OA and control groups (p = 0.001–0.036). The selected AE signals, KI, age and BMI were used to construct a predictive model for radiographic OA with AUC of 90.3% (95% CI 83.5–97.2%) which showed a statistical improvement of the reference model based on age and BMI, with AUC of 84.2% (95% CI 74.8–93.6%). In males, the predictive model failed to improve the reference model. AE and KI provide complementary information to detect radiographic knee OA in females.

## Introduction

Osteoarthritis (OA) of the knee is a common disease and a major public health issue with increasing prevalence worldwide^1^. OA often causes pain, restricts mobility, and is considered as the fundamental cause of loss of function in older people affecting 19.2–27.8% of people over 45 years of age^2^. Furthermore, local inflammatory component is often involved in OA^3^, and OA is considered a complex joint disorder with multiple risk factors. The diagnosis of OA is based on patient history, physical examination, and imaging. Currently, conventional radiography is considered the gold standard and the most widely used imaging technique. As complementary modalities, magnetic resonance imaging (MRI), ultrasonography (US) and computed tomography (CT) can also be used to diagnose OA on some occasions^4^. However, the clinical diagnosis of osteoarthritis is often problematic since the phenotype of OA is variable, and only a poor correlation between clinical and imaging findings exists^5^.

In addition to conventional imaging, some studies have indicated a potential of using acoustic emissions (AE)^6–10^ and kinematic instability (KI)^11–14^ as biomarkers for cost-efficient diagnosis. Early detection of OA could be achieved by measuring AE from the knee with the rationale that smooth, optimally lubricated cartilage surfaces slide tacitly against each other, whereas rugged, suboptimally lubricated surfaces move unevenly, generating more acoustic signals^8^ commonly referred as *crepitus*. AE are generated by transient elastic waves produced by a sudden redistribution of stress in a material and can be recorded from the surface of the knee. It has been demonstrated in vitro that AE are associated with cartilage damage in animal model^15^, and several studies have shown that AE could be applied to detect OA of the knee joint^8–10,16^. Furthermore, it has been reported that OA is associated with sensations of knee joint instability, such as buckling, shifting or giving away of the joint^12^, but also with activity limitations^14^. Gait analysis has been suggested as a potential method to quantify the information occurring during walk and especially detect abnormal movement. Measured parameters from the gait analysis have been considered as suitable objective markers of kinematic instability^13^. However, while the previous studies have been focusing on video-based approaches, the recent developments of inertial measurement units (IMU) allow to evaluate similar information using embedded sensors into wearable devices. The advantage of these wearable solutions is to provide a low-cost and easy-to-use modality, providing spatial information of the segment of the limb studied.

As the prior studies have examined the applicability of these biomarkers separately, here we aimed to combine these signals to detect radiographic knee OA. This is essential as radiography causes radiation burden and poses issues to detect early-stage OA; furthermore, equipment needed for radiography are expensive, regulated and operating them requires specialized personnel. Accordingly, the combined biomarkers could lead to early detection of knee OA diminishing the need for radiography. Therefore, the purpose of this study was to evaluate the AE and KI signals of the osteoarthritic knee joints and to compare these signals to the corresponding radiographic findings.

## Results

At first, AE signals were processed, and multiple statistical measures were applied. Receiver operating characteristic (ROC) curves were then used to pick the best signals with a threshold of AUC > 0.600 (Tables 1 and 2), and LOO cross validation was used to make predictive models. In female subjects, AE medial extension ratio (high frequency and clicks) (*p* = 0.005), AE lateral flexion kurtosis (high frequency) (*p* = 0.036), KI (*p* < 0.001), BMI (*p* < 0.001) and age (*p* = 0.005) had a significant statistical difference between the OA and control groups. In male subjects, AE lateral sit-to-stand skewness (low frequency) (p = 0.034), AE lateral sit-to-stand kurtosis (low frequency) (p = 0.036) and BMI (p = 0.032) had a significant statistical difference between the OA and control groups.

**Table 1:** AUC values of the best test result variables in females.

Signal	AUC	Signal	AUC	Signal	AUC
BMI	0.846	AE lateral extension ratio (hf and cl)	0.652	AE medial sit-to-stand skewness (lf)	0.624
Kinematic instability	0.796	AE medial extension ratio (hf and lf)	0.647	AE medial sit-to-stand kurtosis (lf)	0.612
AGE	0.687	AE medial sit-to-stand ratio (hf and cl)	0.642	AE medial sit-to-stand ratio (hf and lf)	0.611
AE medial extension ratio (hf and cl)	0.670	AE lateral extension ratio (hf and lf)	0.637	AE lateral sit-to-stand skewness (hf)	0.608
AE lateral flexion kurtosis (lf)	0.670	AE lateral sit-to-stand skewness (lf)	0.627	AE lateral sit-to-stand kurtosis (all)	0.605
AE lateral flexion skewness (lf)	0.666	AE lateral sit-to-stand kurtosis (lf)	0.625	AE lateral sit-to-stand skewness (all)	0.605
AE lateral flexion kurtosis (hf)	0.659				

*AE* acoustic emission, *BMI* body mass index, *hf* high frequency, *lf* low frequency, *cl* clicks, *all* high and low frequency.

**Table 2:** AUC values of the best test result variables in males.

Test result variable(s)	AUC
AE lateral sit-to-stand skewness (lf)	0.661
AE lateral sit-to-stand kurtosis (lf)	0.654
AGE	0.640
BMI	0.639
AE medial extension skewness (hf)	0.633
AE lateral sit-to-stand ratio (hf and lf)	0.624
AE lateral flexion ratio (hf and lf)	0.622
AE lateral flexion ratio (hf and cl)	0.622
AE lateral extension skewness (hf)	0.620
AE lateral sit-to-stand ratio (hf and cl)	0.607
Kinematic instability	0.600

*AE* acoustic emission, *BMI* body mass index, *hf* high frequency, *lf* low frequency, *cl* clicks, *all* high and low frequency.

Subsequently, we assessed whether the selected signals could differentiate between the presence or absence of specific OA findings. Out of the selected signal, particularly the AE signals assessed medially during flexion–extension and the KI showed good potential to detect OA changes (*p* = 0.001–0.043) in female subjects. In males, the selected signals showed rather poor diagnostic performance. Tables 3 and 4 show the statistically significant *p-*values, when the selected signals were used to detect knee OA changes in females and males, respectively.

**Table 3:** The ability of acoustic emissions and kinematic instability (AUC > 0.600) to detect specific osteoarthritic changes in females.

Signal	Medial joint space narrowing (no = 34/yes = 32)	Lateral joint space narrowing (no = 59/yes = 7)	Medial femoral osteophytes (no = 51/yes = 15)	Medial tibial osteophytes (no = 30/yes = 36)	Lateral femoral osteophytes (no = 57/yes = 9)	Lateral tibial osteophytes (no = 40/yes = 26)
Age						p = 0.004
BMI	p = 0.009		p = 0.003	p < 0.001	p < 0.001	p = 0.001
AE medial extension ratio (hf and lf)						
AE medial extension ratio (hf and cl)	p = 0.032		p = 0.040	p = 0.001	p = 0.043	p = 0.001
AE lateral extension ratio (hf and lf)	p = 0.032					p = 0.015
AE lateral extension ratio (hf and cl)						p = 0.039
AE lateral flexion kurtosis (hf)						
AE lateral flexion skewness (lf)						
AE medial sit-to-stand ratio (hf and lf)						
AE medial sit-to-stand ratio (hf and cl)	p = 0.006			p = 0.007		
AE medial sit-to-stand kurtosis (lf)						
AE medial sit-to-stand skewness (lf)						
AE lateral sit-to-stand skewness (hf)		p = 0.039				p = 0.013
AE lateral sit-to-stand kurtosis (all)		p = 0.007				p = 0.018
AE lateral sit-to-stand kurtosis (lf)						
AE lateral sit-to-stand skewness (all)		p = 0.003				p = 0.022
AE lateral sit-to-stand skewness (lf)						
Kinematic instability			p = 0.002	p < 0.001	p = 0.018	p = 0.001

The absence or presence of osteoarthritic changes on conventional radiography are given in parentheses (no/yes), and corresponding statistically significant p-values for each biomarker.

*AE* acoustic emission, *BMI* body mass index, *hf* high frequency, *lf* low frequency, *cl* clicks, *all* high and low frequency.

**Table 4:** The ability of acoustic emissions and kinematic instability (AUC > 0.600) to detect specific osteoarthritic changes in males.

Signal	Medial joint space narrowing (no = 22/yes = 21)	Lateral joint space narrowing (no = 39/yes = 4)	Medial femoral osteophytes (no = 36/yes = 7)	Medial tibial osteophytes (no = 18/yes = 25)	Lateral femoral osteophytes (no = 39/yes = 4)	Lateral tibial osteophytes (no = 29/yes = 14)
Age						
BMI						p = 0.008
AE medial extension skewness (hf)				p = 0.013		
AE lateral flexion ratio (hf and lf)						
AE lateral extension skewness (hf)						
AE lateral flexion ratio (hf and cl)						
AE lateral sit-to-stand ratio (hf and lf)						
AE lateral sit-to-stand ratio (hf and cl)						
AE lateral sit-to-stand skewness (lf)	p = 0.007					
AE lateral sit-to-stand kurtosis (lf)	p = 0.007					
Kinematic instability		p = 0.040				

The absence or presence of osteoarthritic changes on conventional radiography are given in parentheses (no/yes), and corresponding statistically significant p-values for each biomarker.

*AE* acoustic emission, *BMI* body mass index, *hf* high frequency, *lf* low frequency, *cl* clicks, *all* high and low frequency.

**Figure 1:**
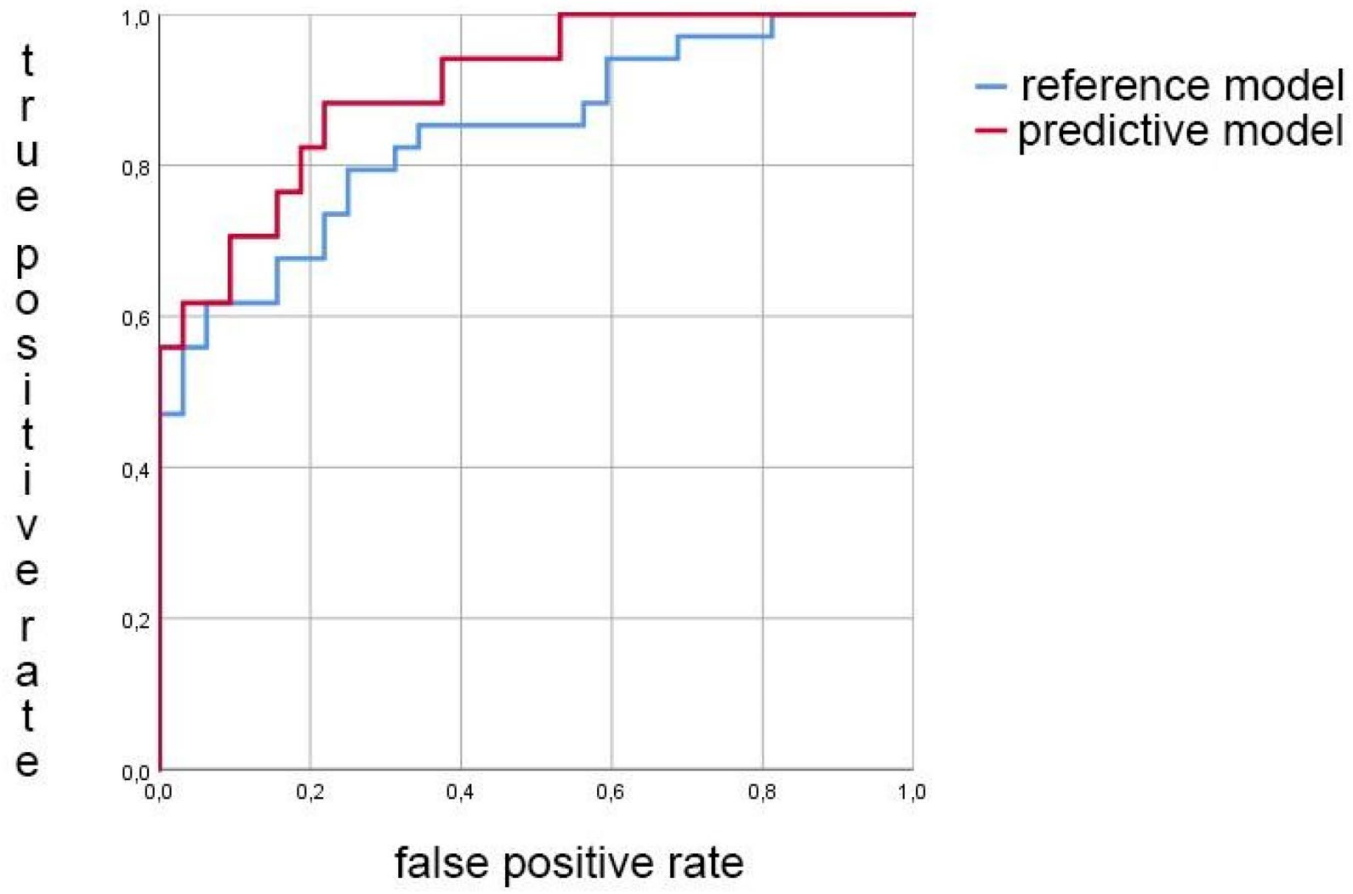
The receiver-operator curves (ROCs) for the predictive model (red) using selected AE signals, KI, age and BMI, and for the reference model (blue) using age and BMI in females. Significant statistical difference was found using DeLong method (*p* < 0.001).

**Figure 2:**
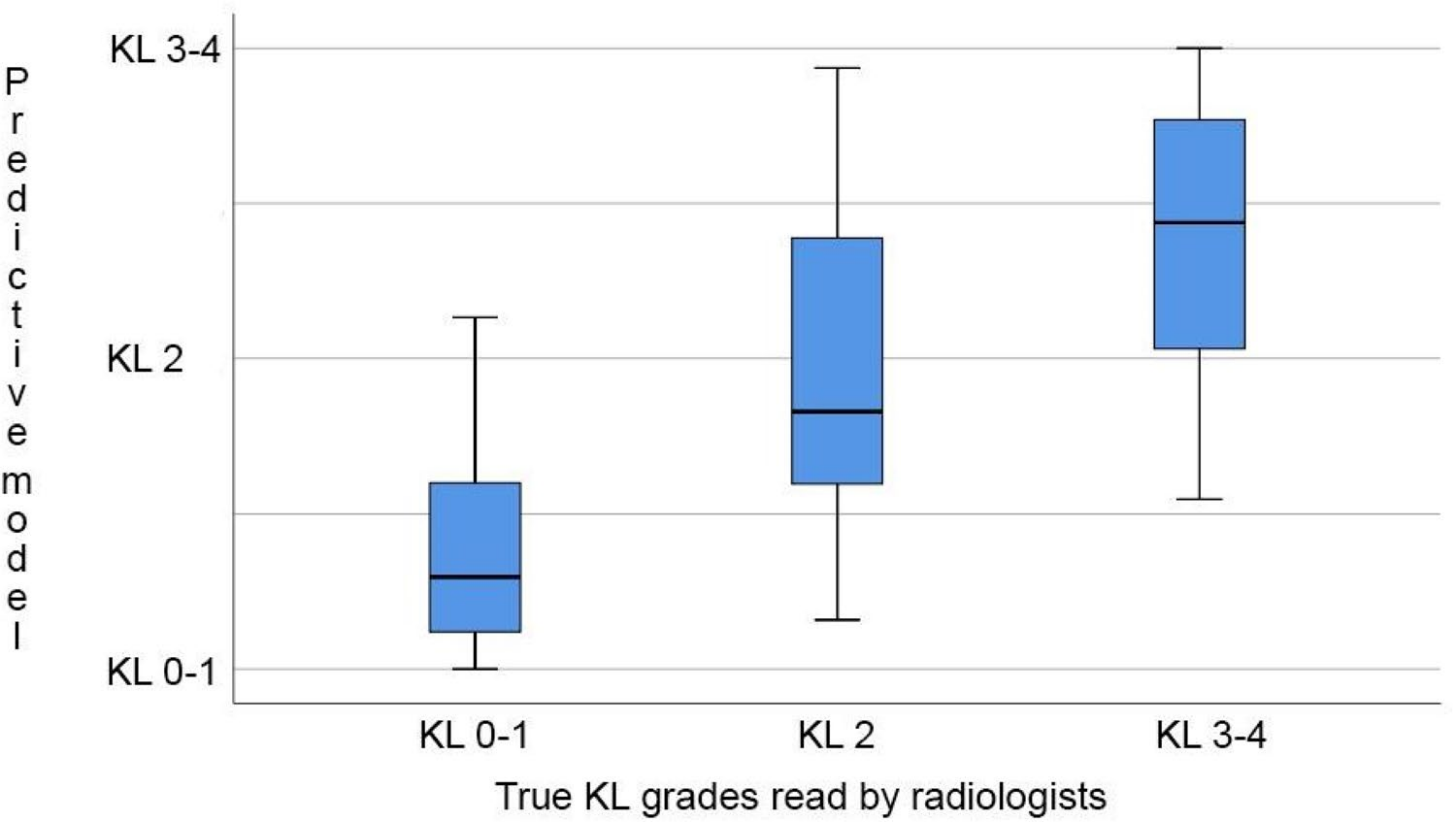
The boxplot describing the predicted KL grades (Y-axis) according to the predictive model within the radiologic KL grades (X-axis) in female subjects.

In female subjects, selected AE signals, KI, age and BMI were used to build a predictive model with an AUC of 90.3% (95% CI 83.5–97.2%). When only age and BMI were used in a reference model, the AUC was 84.2% (95% CI 74.8–93.6%); moreover, there was a statistical difference between these models (Fig. 1). When performing ROC analysis with only KI, the AUC was 76.2%, while combined with BMI and AGE, the AUC was 87.2%. For the best AE signals, the AUC was 77.3%. Subsequently, we investigated whether our models correlated with severity of the OA according to KL grading; for the predictive model the Spearman correlation coefficient was 0.724 (*p* < 0.001), and for the reference model it was 0.625 (*p* < 0.001). Figure 2 shows boxplot presentation of the predicted KL grades within the evaluated radiographic KL grades in females.

**Figure 3:**
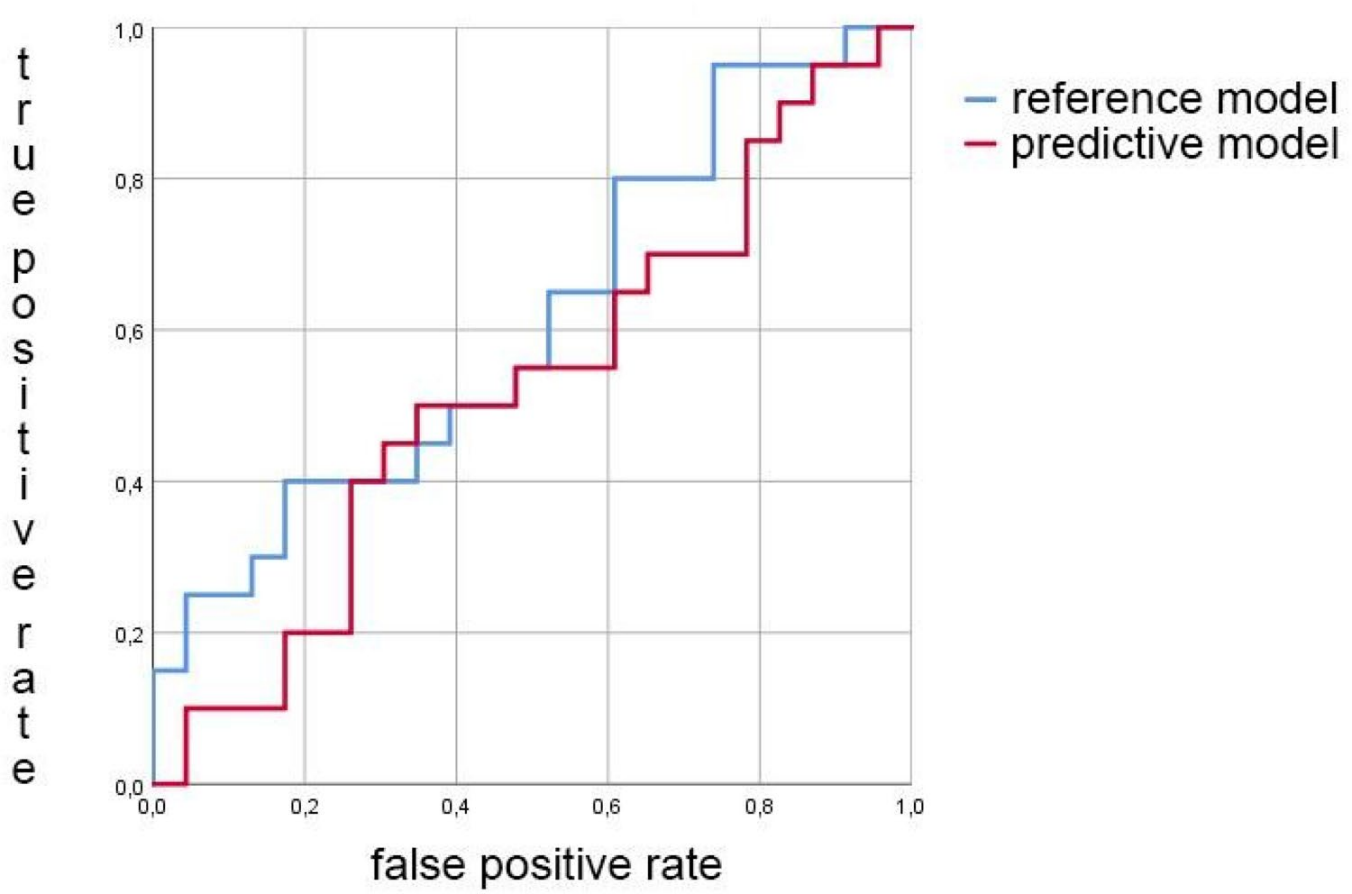
The receiver-operator curves (ROCs) for the predictive model (red) using selected AE signals, KI, age and BMI, and for the reference model (blue) using age and BMI in males. No significant statistical difference was found using DeLong method (*p* = 0.36).

**Figure 4:**
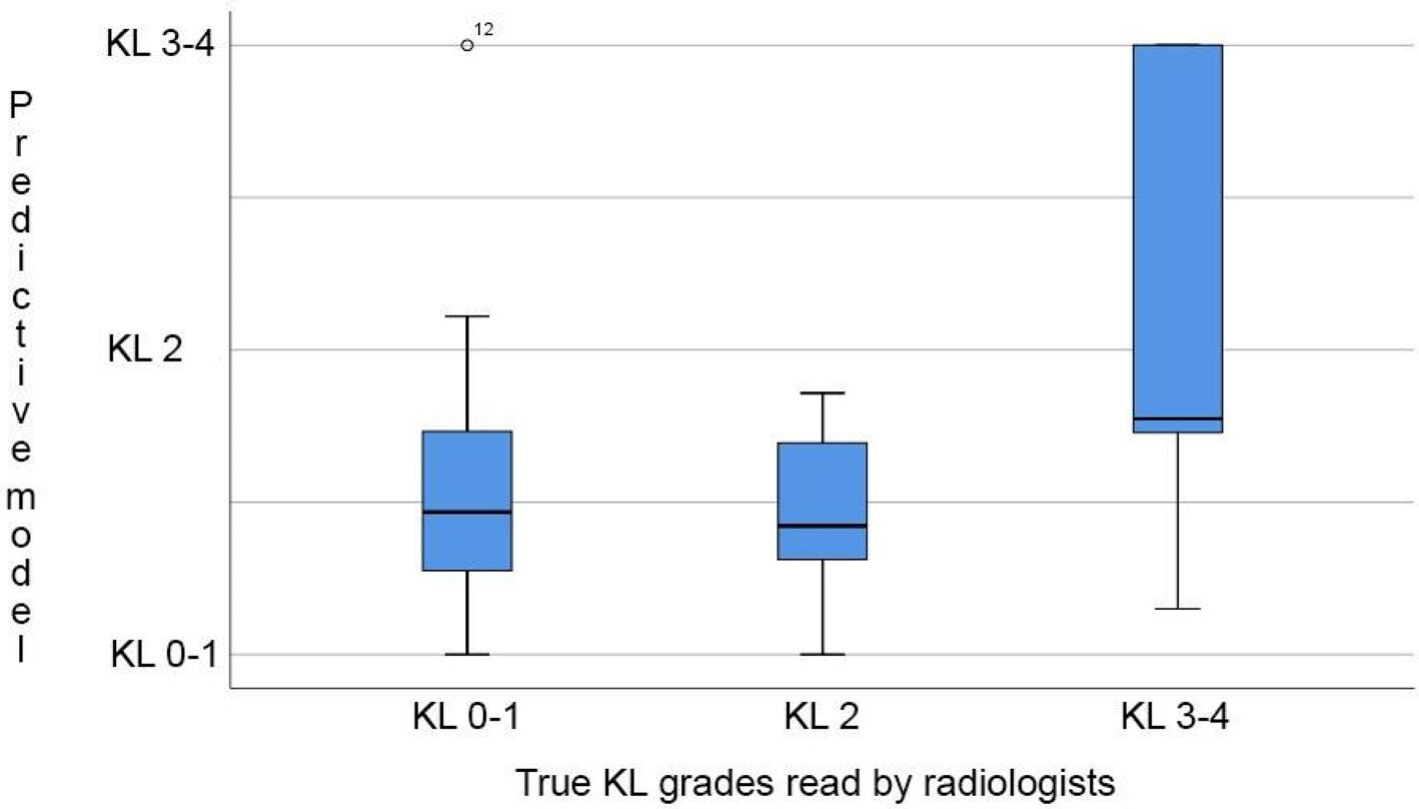
The boxplot describing the predicted KL grades (Y-axis) according to the predictive model within the radiologic KL grades (X-axis) in male subjects.

In male subjects, selected AE signals, KI, age and BMI were used to produce a predictive model with an AUC of 52.6% (CI 33.5–68.5%). When only age and BMI were used in a reference model, the AUC was 61.1% (CI 42.2–76.7%); no statistical difference was found between the models (p = 0.36) (Fig. 3). Spearman correlation coefficient with KL grading was 0.157 (*p* = 0.313) for the predictive model, and 0.269 (*p* = 0.082) for the reference model. Figure 4 depicts boxplot of the predicted KL grades within the evaluated radiographic KL grades in males.

## Discussion

In this study, our objective was to evaluate the acoustic emissions and kinematic instability of the osteoarthritic knee joints, and compare these signals to radiographic findings. We have shown the potential benefit of both acoustic and kinematic modalities to detect knee OA. When combining the selected AE signals, KI, age and BMI in female subjects, we were able to produce a predictive model with an AUC of 90.3% suggesting that the admixture of these biomarkers could be applied as a complementary tool for OA diagnostics. The main benefit of the approach presented here is the combination of multiple modalities in a wearable device, offering the opportunities for further developments of novel low-cost supporting tools for the evaluation of knee joint integrity.

In a large study applying the Osteoarthritis Initiative (OAI) data with over 3000 knees, subjective crepitus of the knee joint was shown to predict incident symptomatic OA longitudinally^7^. Previously, also AE signals have been used to distinguish between normal and osteoarthritic knee joints successfully^17^. In a recent study consisting of 68 subjects, the authors showed that AE could differentiate normal knees (KL 1) from OA knees (KL 2–4) during sit-to-stand tests; however, within the OA knees no statistical differences between the subgroups could be detected^9^. In another study, in a small sample of seven OA knees and seven healthy controls, AE signals were significantly higher in the OA group^8^. Furthermore, Mascaro et al.^16^ reported that knees with OA produce 6–10 times more AE signals than healthy knees, with amplitudes which can be 20 dB higher, and durations which can be ten times longer; however, the study was hindered by a small data set of 11 healthy knees and 10 OA knees using sit-to-stand tests. On the technical aspect, it has been implicated that AE can be applied as a biomarker to monitor joint ageing and OA^10^; later on, Töreyin et al.^18^ verified that AE consistency can be quantified in ambulatory subjects performing every-day activities and showed that surrounding noise during the AE measurements does not cause significant interference.

In our study, the acoustic parameters used to discriminate OA subjects were representative of the consistency of the sound’s patterns. In brief, it suggests that knees with inconsistent AEs along similar movements have more likely a rough/damaged cartilage surface, as suggested by random “clicks”. This approach contrasts to most earlier studies counting solely the occurrence of acoustic events (each time the signal crosses a fixed threshold). Furthermore, the correlation between the severity of the condition and the combination of the calculated parameters is in accordance with our previous in vitro study^15^ in which AE signals were associated with cartilage damage severity.

KI is a common finding within knee OA with studies observing prevalence rates of more than 60% for self-reported instability^12^. Unfortunately, the evaluation of instability is based mainly on subjective self-reporting. Parameters measured with gait analysis have been studied as more objective indicators of instability; however, a recent review concluded that although many different candidates for an objective knee stability gait parameter are found in literature, all of them lack sufficient clinical evidence^13^. Here, we assessed the knee instability from one-leg-stand analysis, which we previously reported was associated with changes in morphological features of the cartilage assessed by radiography and MRI^19^. Chaudhari et al.^20^ stated that in subjects suffering from late-stage OA, knee extension strength and pain are associated with perceived KI. The authors concluded that predominant lateral laxity and perceived knee instability are independently associated with unsatisfactory outcomes in people with knee OA^20^.

One main limitation of this study is the distribution of subjects between the groups. As already discussed, the OA group had a BMI and age significantly higher than the control group, resulting in a baseline model reaching already AUC = 84.2% for the discrimination of subjects with KL ≥ 2. While adding acoustic and kinematic information provided a statistically significant improvement of the prediction capability, the increase in terms of percentages was still limited. This raises the question of the necessity of using extra supporting diagnostic tools in cases of overweight elderly subjects; however, the same is true for knee radiography which will still be performed in such cases to confirm the diagnosis. The difference in knee morphology between subjects with low and high BMIs affected the robustness of data acquisition of the acoustic sensors located in the patella part. While different sizes for the shank and thigh part were available, only one patella part size was developed, resulting in extra artefacts due to mechanical restrictions of the device. Another potential limitation of this study is the use of KL grades as a reference. While this standard examination is commonly used for severity assessment, it does not provide direct information on the cartilage damage beside its thinning, whereas the AEs assesses the roughness of the cartilage surface. While from a clinical perspective, this information is complementary to what is available in practices non-invasively, the association with the radiographic features of the KL grades are indirect. While no study to establish the reliability was performed during trial, similar sensors were used in our previous acoustic study^15^ correlating non-contact acoustic emission to cartilage damage, and our kinematic study^19^ correlating compartmental damage to the instability parameter reported here. Furthermore, the prototype was built in rigid material with a focus on fitting the anatomical shape, to increase the robustness of the positioning. Statistical bias may also be present in this study, but the strict correction can be bad or even deleterious in worst scenarios; to tackle these issues we used the leave-one-out technique to strengthen our analyses. Last, it is troublesome that the apparatus failed to improve OA diagnosis in male subjects; the reasons for this remain rather elusive and we can only speculate if the low number of subjects or the fewer OA findings in male group in general were the contributing factors behind this. As OA is a multifactorial disease, a plethora of variables may be affecting our results between the sexes.

Here we have presented a novel approach for the detection of OA with a smart wearable. This study evaluated the potential of such technology to provide new information on the joint integrity, complementary to what is available in clinical practices. The preliminary results reported here suggest the need to further investigate and develop this technology, prior to be validated in a larger cohort.

## Methods

### Subjects

For this single institution case control study, Institutional Review Board approval was obtained (Northern Ostrobothnia Hospital District, Oulu University Hospital; EETTMK 7/2016) and written informed consent was obtained from all subjects. All procedures performed in studies involving human participants were in accordance with the ethical standards of the institutional and/or national research committee and with the 1964 Helsinki declaration and its later amendments or comparable ethical standards. This study was registered at clinicaltrials.gov with study identifier NCT02937064.

Sixty-six female subjects (57.8 ± 6.2 years old, range 44–67 years) and 43 male subjects (58.6 ± 5.5 years old, range 45–64 years) were enrolled in this study. The inclusion criteria were age of 45–65, and the exclusion criteria rheumatoid arthritis, weight over 110 kg, and previous total knee replacement surgery. The subjects with OA were recruited using patient records of our institution and subjects without OA were recruited using a newspaper advertisement. Based on radiography, 54 subjects (49.5%) were diagnosed with radiographic knee OA (Kellgren–Lawrence grade ≥ 2) forming the study group, and the remaining 55 subjects (50.5%) with KL grade < 2 formed the control group. For the analyses, the female subjects (34 OA cases, 32 controls) and the male subjects (20 OA cases, 23 controls) formed their own subgroups.

### Apparatus for signal collection

**Figure 5:**
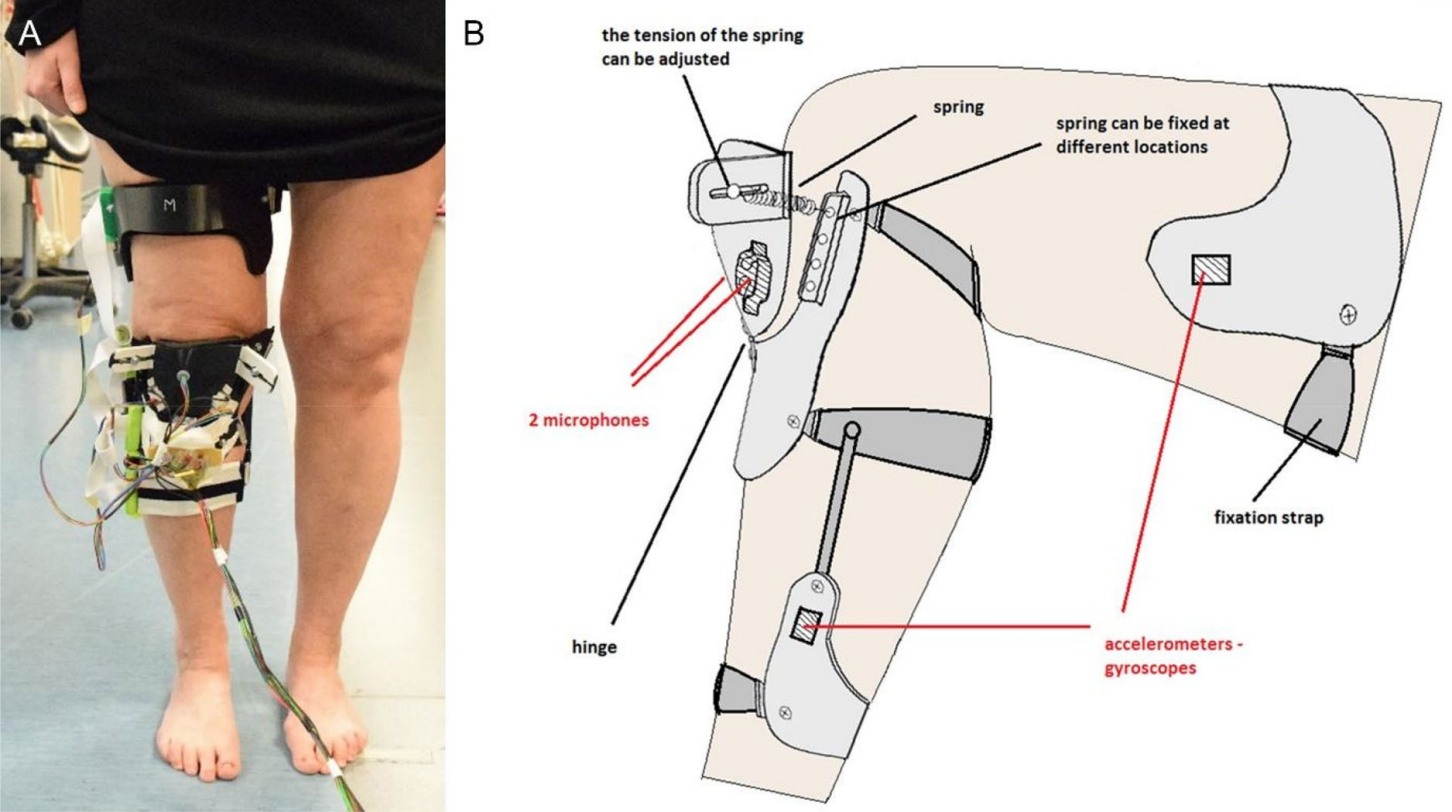
The apparatus used to collect the acoustic emission and kinematic signals on a test subject (**A**). The raw schematics of the device are also shown (**B**).

A custom-made apparatus similar to a knee orthosis was developed to allow the reproducibility of the sensors’ location used for data acquisition, without hindering the movement of the subject. The design of the device was created using FEMAP software, and was 3D printed in polylactide (PLA) using a Prusa MK3 printer. Furthermore, a resin printer (Formlabs Form 2) was used for all the sensor frames embedded in the apparatus, as they required higher printing resolution for an optimal fit. An ethylene vinyl acetate (EVA) padding foam covered all the parts of the apparatus in contact with the skin of the subject. The overall apparatus was made of three main parts attached to the limb of the subject with the help of straps: thigh, shin and patella parts. The acoustic signals were measured from both medial and lateral sides of the knee joint using non-contact air microphones (Audio-Technica^®^ AT899) with a sampling frequency (SF) of 44.1 kHz and a soundcard (Focusrite-Scarlett 18i8 18) with frequency of 192 kHz. IMU sensors (SparkFun 6 DOF IMU Digital Combo Board—ITG3200/ADXL345) applying a frequency of 100 Hz using an I2C interface were placed on thigh and shin respectively. The accelerations from the 3 tri-axial accelerometer were used both for segmentation of acoustic sensors and assessment of kinematic instability. Figure 5 demonstrates the appearance of the apparatus.

### Acquisition protocol

The subjects were invited to our institution, in the radiographic outpatient ward. After filling a consent form, changing to shorts and removing shoes, the subjects had height and weight measurements. They filled pain surveys and were photographed while in a comfortable standing position. The device was then installed by a nurse who instructed the subjects prior to each exercise. Subjects performed the following simple tasks while wearing the device in order to record AE and KI of the knee: ten times flexion–extension (FE) of the knee, ten times sit-to-stand (STS) tests to assess knee friction, and two times one-leg-stands to evaluate KI. Two researchers were present in the room to make sure that all the measurements were correctly collected. During the acquisition of acoustic signals, all the persons in the room were silent to reduce artefact noises. Following signal acquisition, the prototype was removed by the nurse and the subjects were sent to radiographic imaging.

### Signal analysis

As pre-processing, the flexion and extension phases of the acoustic signals were segmented based on the kinematic information collected by the accelerometers. The selected signal parts were then filtered in the range of 5–20 kHz using a 5th order Butterworth filter and decimated four-times to reduce acoustic signal redundancy. Eventually, we searched AE candidate locations using a root-mean-square thresholding approach. We removed duplicates by grouping candidates with close locations. An average click pattern was derived from windows of 5 ms centered in each identified AE location. We computed a cross-correlation spread using the Gaussian fit and used its width, σ, as a similarity measure between each particular AE and the average pattern. Finally, we analysed the number of clicks below the threshold σt = 1.5 ms (most similar to the average pattern) divided by the total number of threshold crossings.

For the KI evaluation, the acceleration in anatomical longitudinal axis was used as a measure of KI and the power of the signal over the two repeated movements was calculated, as reported in by Virtanen et al.^19^.

### Imaging technique and interpretation

Standard weight-bearing postero-anterior knee radiography was performed on each subject. The X-ray beam was 10° caudally angulated and the knee was supported by a frame in 20° flexion and foot in 5° external rotation. On knee radiography, joint-space narrowing, osteophytes and Kellgren–Lawrence (KL) grade were evaluated: joint space narrowing was graded medially and laterally as normal or narrowed. Osteophytes were evaluated from medial and lateral aspects of the knee joint both on the femur and tibia and graded as absent or present. Ultimately, the total KL grade (0–4) was given to each knee joint. The specific OA changes were graded by a single radiologist (5 years of experience). The KL grading was performed separately by two radiologists—one with 5 years of experience and another with 20 years of experience; in case of disagreement, third consensus read was performed by the first radiologist.

### Statistical analysis

The data from STS, FE and KI tests were fused in a logistic regression (LR) model and compared to a presence of radiographic OA. We compared the added value of the proposed biomarkers with a reference model, using the area under the receiver operating characteristic curve (AUC) computed by leave-one-out (LOO) cross-validation. The reference model included age and body mass index (BMI) as predictors. Eventually, we added STS, FE and KI parameters one by one as well as their interaction terms and performed the final AUC comparison using DeLong test and bootstrapping. T-test was used to test for association between the collected signals and specific OA changes, and Spearman correlation analysis to correlate our models with the severity of the OA according to KL grading.

## Data Availability

The datasets generated during and/or analysed during the current study are available from the corresponding author on reasonable request.
